# White phosphorous civilian hand burns – An aid to timely treatment of a rare entity

**DOI:** 10.1016/j.tcr.2024.100983

**Published:** 2024-03-04

**Authors:** Eimear Phoenix, Varit Suwanwalaikorn, Jordan Wilkinson, Colin M. Morrison, Roisin T. Dolan

**Affiliations:** a Department of Plastic and Reconstructive Surgery, St Vincent's University Hospital, Dublin, Ireland; b Royal College of Surgeons Ireland, St Stephen's Green, Dublin, Ireland

**Keywords:** Chemical burns, Hand burns, Hand trauma, upper limb trauma

## Abstract

First discovered in 1669, white phosphorus is well known for its use in military warfare (Davis, 2002). Its application has since been expanded to include industrial disinfectants, fertilisers and fireworks (Davis, 2002). Exposure to white phosphorus can lead to severe chemical burns with high morbidity and potentially fatal systemic effects. Fortunately, civilian casualties from this potent agent are remarkably rare with few reports in the literature to date (Frank et al., 2008; Aviv et al., 2017). We present the case of a 27-year-old fisherman who sustained a chemical burn to his right hand from a substance suspected to be white phosphorus. We propose an evidence-based algorithm to guide non-military physicians literature on the acute management of white phosphorus burns to optimise timely emergency management of this uncommonly encountered substance.

## Background

White phosphorus is a toxic, synthetic material first discovered in 1669 by German Chemist Hennig Brandt during his quest for the ‘Philosopher Stone’, a legendary substance that could convert base metals into gold [3]. Its uses in military warfare as part of explosive artilleries and smoke-producing munitions in both World War I and II are well documented [1]. Military personnel are at high risk of phosphorus burns from their use in incendiary shells and detonators, resulting in higher surface area burns than those reported in civilians.

White phosphorus is produced by heating pellets of phosphate rock with coke or silica in an electric furnace [4]. The phosphorus vapor is then purified and collected by passing the vapor through an electrostatic precipitator and condenser. White phosphorus has a melting point of 44 °C, making it insoluble in water [4]. However, it is extremely reactive in the present of atmospheric oxygen. White phosphorus burns result from both a chemical and thermal reaction. Phosphorus reacts with oxygen producing phosphorus pentoxide during a thermal reaction. Chemical injury results from the corrosive action of phosphoric acids which are formed during combustion.

We present the case of a 27 years old fisherman whom sustained a 1 % total body surface area (TBSA) chemical burn of his right hand after he came in contact with a substance suspected to be white phosphorus whilst working on an industrial fishing boat.

## Case presentation

**Figs. 1 & 2:**
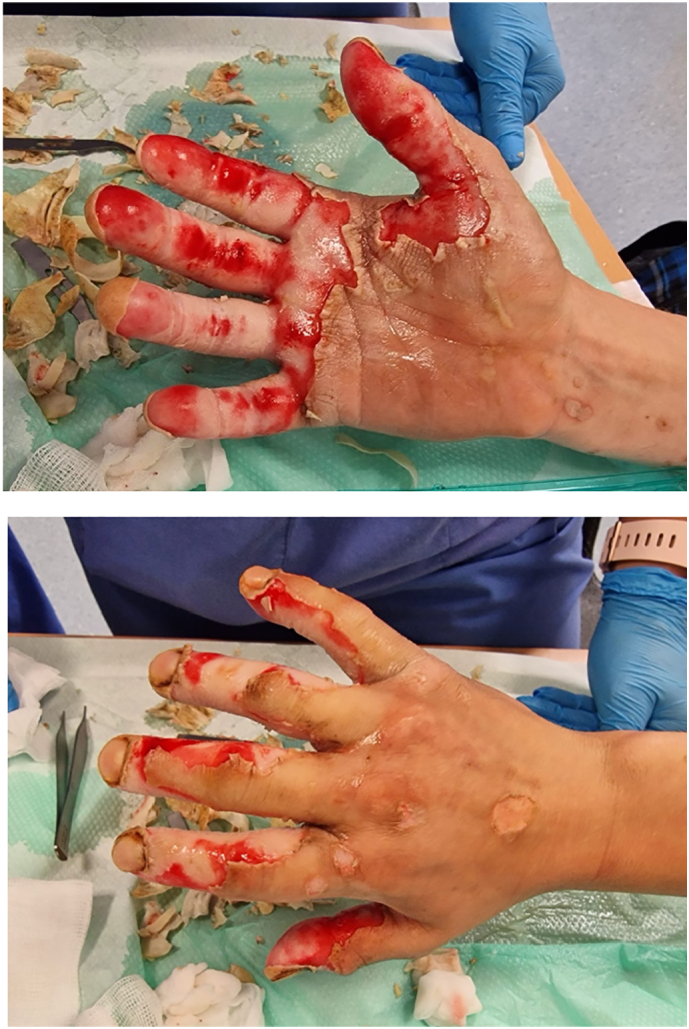
Clinical photographs demonstrating mixed thickness burns predominately affecting the palm and fingers of his right hand.

A 27 year old right hand dominant fisherman presented to our emergency department with a chemical burn to his right hand [Figs. 1 & 2]. The injury occurred 16 h prior on a fishing trawler whilst at sea. As he pulled in his fishing net he reports a ‘smoking white block’ which he removed from his net without use of protective gloves. He reports holding the object briefly before discarding it overboard. He initially bathed his hands in sea water followed by cold tap water for approximately 30 min. He wrapped his hand in a dry dressing and presented to the emergency department once the ship arrived on shore.

On arrival to the emergency department the patient was haemodynamically stable. Assessment revealed a less than 1 % mixed thickness burn to his right hand. The skin pH was 11 and he underwent initial copious irrigation with normal saline by the emergency physicians. An electrocardiogram was performed which showed sinus rhythm and his electrolytes were also normal. Plastic surgery were consulted and he was transferred urgently to the operating room for further management.

**Figs. 3 & 4:**
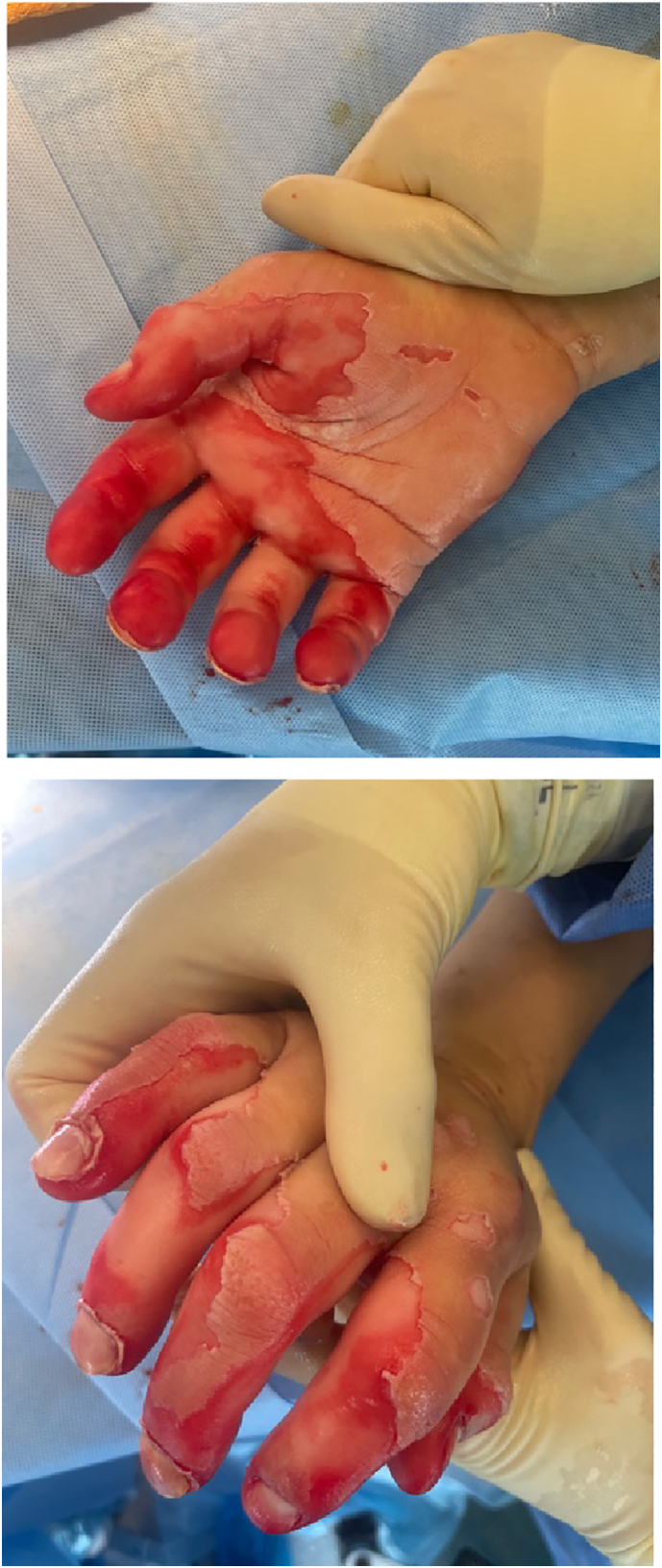
Intra-operative images following our patient's first surgical debridement & washout.

In the operating theatre, all non-viable skin was debrided and he underwent copious washout with 0.9 % saline. His first intraoperative pH was 9 and 14 l of saline were required to achieve a pH of 7. Examination of the burn following washout confirmed a less than 1 % TBSA mixed thickness burn involving the palm, thumb and all digits [Figs. 3 & 4]. His burn was dressed with Jelonet®, gauze, wool and he was placed in a volar resting splint.

**Figs. 5 & 6:**
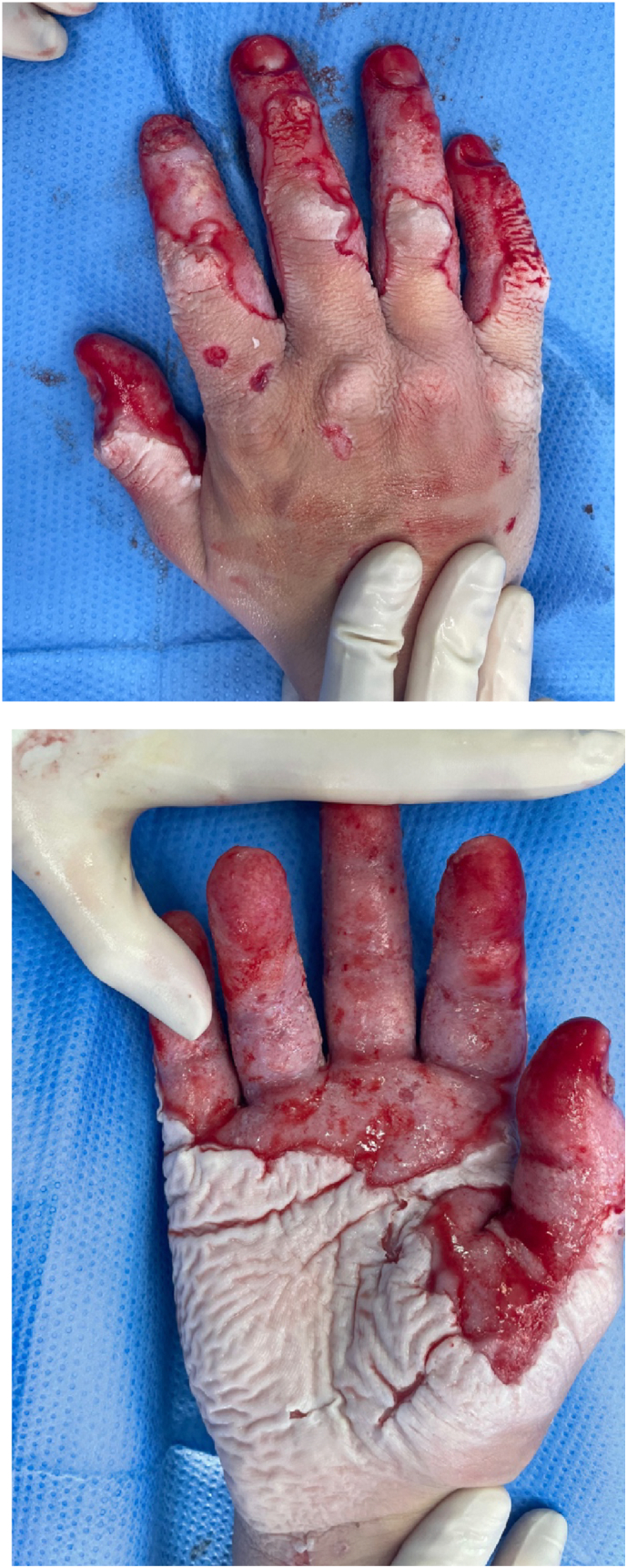
Intra-operative images following 1st change of dressing of right-hand burn.

**Fig. 7:**
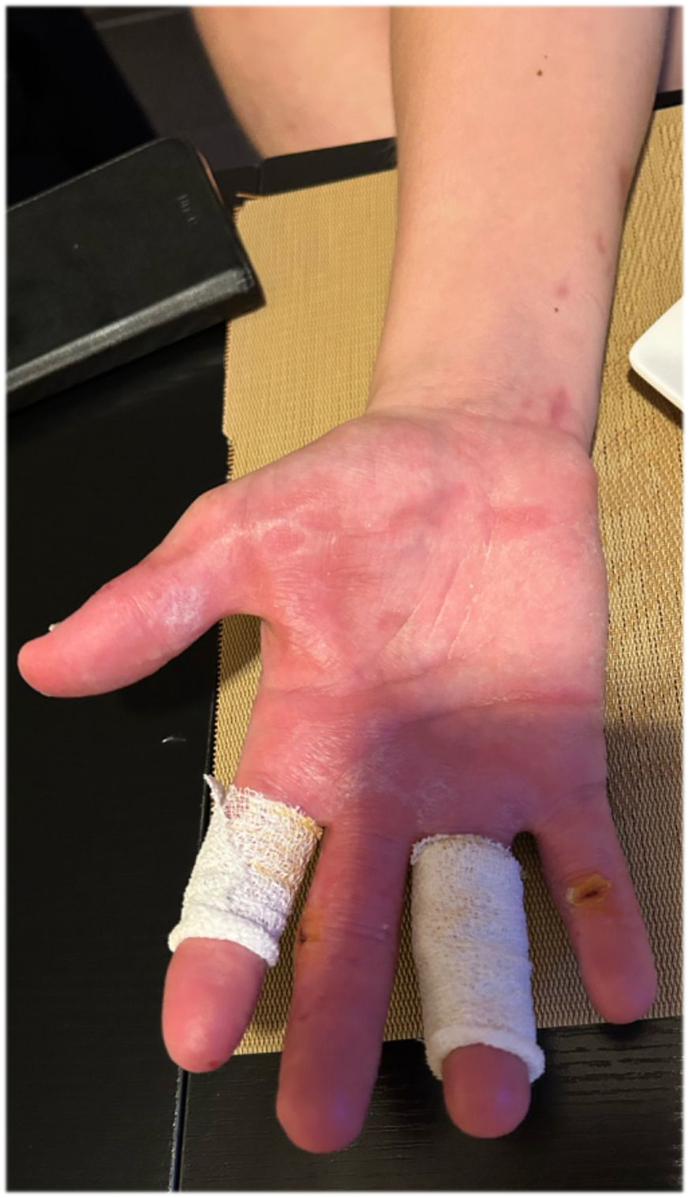
Image provided by the patient 6 months post injury showing the right palm fully healed and minimal dressings remaining on the index and ring fingers.

Two days post-operatively he returned to theatre for first dressing change. Examination under anaesthetic revealed a clean, granulating wound bed with no deeper conversion of the burn and he was redressed with Aquacel Ag® and wraps [Figs. 5 & 6]. Culture swabs from his second procedure cultivated methicillin resistant staphyloccus aureus (MRSA). No signs of clinical infection negated commencement of antibiotics. He underwent 2 further dressing changes at ward level and his dressings were changed to Flamazine bags. He remained well throughout his inpatient stay and on day 13 of admission he was repatriated to a military burns unit in his home country where he continued to be managed with dressings & regular hand therapy. At 6 months following his initial injury, his burn is nearly fully healed and the patient reports acceptable range of movement of all digits and is able to perform most daily activities [Fig. 7].

## Discussion

White phosphorus burns have been reported in the literature since the early 1940s [1,2]. Typically these burns occur in warzones, resulting in large TBSA burns and are managed by military physicians. Systemic effects of white phosphorus are well described, more commonly occurring in military cohorts. Fat soluble burns from white phosphorous can result in necrosis to the liver and kidneys, which may lead to multiorgan failure [4]. Hypocalcaemia and hypophosphatemia with potential sequalae of cardiac arrythmias are also well documented. Karunadasa et al. report 2 cases of soldiers who sustained large TBSA white phosphorus burns from a grenade blast, resulting in electrolyte abnormalities, cardiac arrythmia and systemic instability [5].

Case reports in civilians are exceptionally rare and are more likely to result in smaller surface area burns. Aviv et al. report the case of a holidaymaker who mistook white phosphorus on a German beach for quartz and sustained 2 % mixed thickness burns to both hands [2]. Similar reports are described by Frank et al. of civilians who mistook the rock for precious stone and stored it in their pockets, sustained full thickness chemical burns to the lateral thigh [1]. In the former case, the presence of white phosphorus was confirmed by chemical testing from sediment found on the patient's clothing. A limitation of our report is the inability to confirm that the substance that caused our patient's burn was in fact white phosphorus. However, the authors highly suspect that this burn was indeed caused by white phosphorus based on the description offered by the patient and the burn pattern itself.

Acute management of burns secondary to white phosphorus should involve removing all contaminated clothing followed by immediate copious irrigation with normal saline [6]. Phosphorus will continue to burn and damage tissues whilst exposed to atmospheric oxygen, hence all particles should be removed as far as possible followed by copious washout with normal saline. Following lavage, white phosphorus burns should be dressed in saline soaked gauze to prevent further combustion of smaller particles and to dilute residual phosphoric acid [[6], [7], [8]]. This should be applied when transporting patients directly to theatre or transferring patients to a tertiary burns unit. Further irrigation should be undertaken in theatre until a neutral pH is achieved.

Early studies discuss the benefits of copper sulphate in the acute management of burns secondary to white phosphorus [9]. Copper sulphate is not an antidote nor neutralising agent for white phosphorus, but rather it blackens small particles making them easily visible and thus aiding decontamination. However, later studies have shown potential toxic side effects of topical copper sulphate when absorbed systemically. Eldad et al. describe the toxic effect of copper sulphate such as from gastrointestinal upset to haemolysis, hepatic necrosis and multi-organ failure [10]. For such reasons, use of copper sulphate is discouraged and copious irrigation with normal saline should be first line of acute management.

**Fig. 8:**
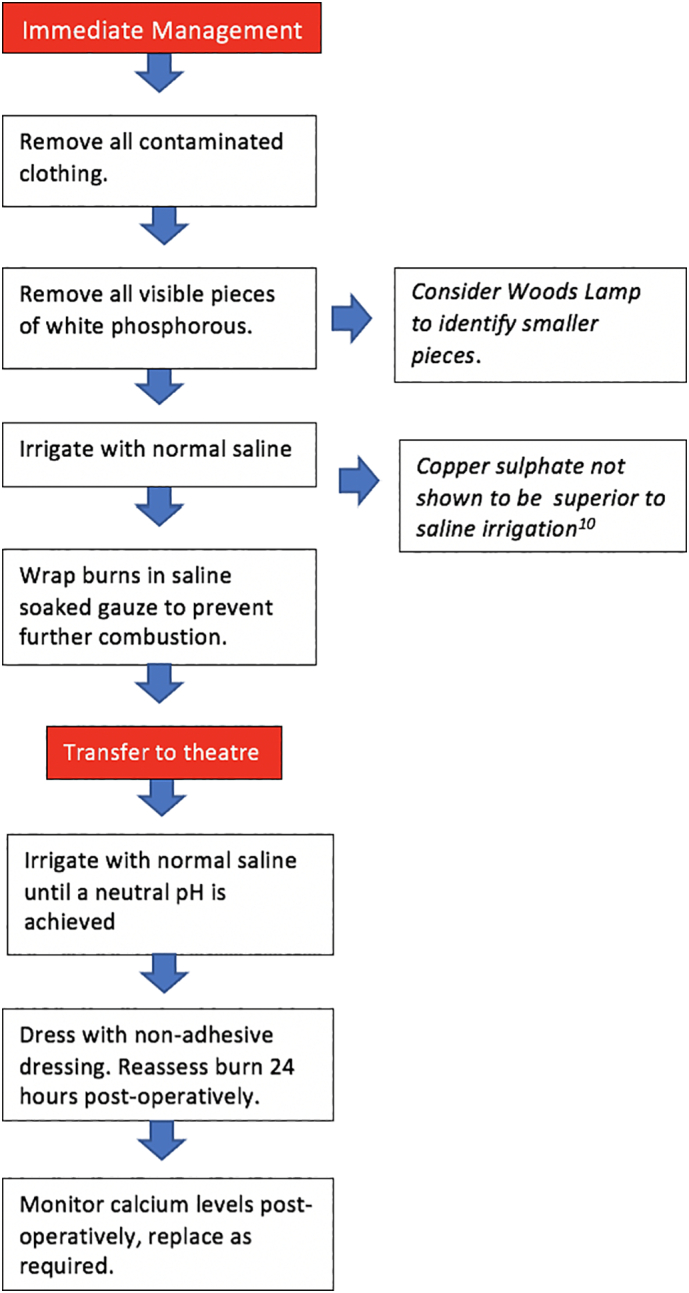
The evidence based immediate and operative management of white phosphorous burns.

We propose an evidence-based algorithm to guide non-military physicians literature on the acute management of white phosphorus burns to optimise timely emergency management of this uncommonly encountered substance [Fig. 8].

## Funding

None.

## Author roles

Eimear Phoenix; main author, literature review.

Varit Suwanwalaikorn; co-author, draft review.

Jordan Wilkinson; co-author, draft review.

Colin M Morrison; senior author, draft review and final approval.

Roisin T Dolan; senior author, draft revision and final approval.

This article has not been presented at any scientific meeting at the time of submission.

## CRediT authorship contribution statement

**Eimear Phoenix:** Writing – original draft, Writing – review & editing. **Varit Suwanwalaikorn:** Writing – review & editing. **Jordan Wilkinson:** Writing – review & editing. **Colin M. Morrison:** Conceptualization, Supervision, Validation, Writing – review & editing. **Roisin T. Dolan:** Conceptualization, Supervision, Writing – review & editing.

## Declaration of competing interest

None.
